# Child safety: a priority within everyone’s reach

**DOI:** 10.1590/1980-220X-REEUSP-2025-0490en

**Published:** 2026-05-11

**Authors:** Luiz Antonio Del Ciampo, Ieda Regina Lopes Del Ciampo

**Affiliations:** 1 Universidade de São Paulo, Faculdade de Medicina de Ribeirão Preto, Departamento de Puericultura e Pediatria, Ribeirão Preto, SP, Brazil.

Dear Editor

The excellent and highly timely article published by Mendes et al. in this journal—whose study aimed to create and validate a serious game mobile app to support the education of caregivers regarding pediatric patient safety measures, targeting the audience of children’s caregivers—prompts reflection on one of the most important health-related issues: child safety. Whether through the creation of a Serious Game mobile app or the opportunity to discuss a serious global public health problem, reading this text highlights the need for programs, initiatives, and mechanisms aimed at enhancing the learning capacity, understanding, and ability of family members and caregivers to take preventive care measures^(1)^. Despite all efforts, so-called unintentional injuries still require greater attention due to their significant impact on children’s health and their social and economic consequences for both families and the community as a whole, as they are a leading cause of long-term complications, increased risk of permanent disability, and death^(2,3)^.

The expansion of research into the epidemiological characteristics of unintentional injuries and a better understanding of their social implications have greatly contributed to the implementation of prevention strategies that incorporate new tools—such as interactive programs and artificial intelligence—to facilitate education as a means of raising awareness, teaching first aid measures, and, above all, promoting preventive actions aimed at addressing the environment and its challenges by modifying them when necessary, as well as guiding ways to adapt human behavior in each situation^(4,5,6)^.

To increase knowledge and awareness among parents and caregivers regarding the characteristics of these injuries and to facilitate understanding of how they occur^(7,8)^, Recognizing the impact on children’s physical and emotional health and development are real opportunities that games can offer the community, by teaching, empowering, and helping to develop and improve skills that can be directed toward greater safety and care for children, always taking into account different social and cultural contexts^(9)^.

## References

[1] Mendes LA, Antunes CC, Sant’Ana MA, Leite Costa ACL, Tourinho FSV, Manzo BF (2025). PEDIATRIX SAFE KIDS: serious Game on the safety of pediatric patients.. Rev Esc Enferm USP.

[2] Jullien S (2021). Prevention of unintentional injuries in children under five years.. BMC Pediatr.

[3] Jin Z, Han B, He J, Huang X, Chen K, Wang J (2023). Unintentional injury and its associated factors among left-behind children: a cross-sectional study.. BMC Psychiatry.

[4] Pollock Z, Draper G, Constantine W, Chalker E, Freebairn L (2024). Injury hospitalisations for children and young people: a 20-year review.. Inj Prev.

[5] Kojima Y (2025). Classification of unintentional injury prevention practices for infants and young children at home: developmental process and associations with other variables in Japanese families.. BMC Psychol.

[6] Sawant DA, Kamble NV (2025). Cross-sectional study of education and knowledge of unintentional injury prevention and postinjury response given by the parents or caregivers of children in Malvani area of Mumbai Suburban District, India.. Inj Prev.

[7] Safe Kids Worldwide (2015). Home safety tips..

[8] Perrin EM, Skinner AC, Sanders LM, Rothman RL, Schildcrout JS, Bian A (2024). The Injury Prevention Program to reduce early childhood injuries: a cluster randomized trial.. Pediatrics.

[9] Chiu RMY, Chan DKC (2024). Understanding parental adherence to early childhood domestic injury prevention: a cross-cultural test of the integrated behavior–change model.. Behav Sci.

