# Supplementary material for: Child safety: a priority within everyone’s reach
Source: Rev Esc Enferm USP. 2026 May 11;60:e20250490. doi: 10.1590/1980-220X-REEUSP-2025-0490en (PMC13163152; doi:10.1590/1980-220X-REEUSP-2025-0490en)
Supplement: Translation

# Translation: Segurança da criança: prioridade ao alcance de todos

**DOI:** 10.1590/1980-220X-REEUSP-2025-0490pt

**Authors:** Luiz Antonio Del Ciampo, Ieda Regina Lopes Del Ciampo

**Affiliations:** 1 Universidade de São Paulo, Faculdade de Medicina de Ribeirão Preto, Departamento de Puericultura e Pediatria, Ribeirão Preto, SP, Brasil.

Prezado Editor,

O excelente e muito oportuno artigo publicado por Mendes et al nesta Revista, cujo estudo teve como objetivo criar e validar um aplicativo móvel de Serious Game para o apoio à educação dos acompanhantes acerca das ações de segurança do paciente pediátrico, dirigido ao público-alvo de acompanhantes de crianças, remete à reflexão sobre uma das mais importantes questões relacionadas à saúde, que é a segurança da criança. Seja pela criação de um aplicativo móvel de Serious Game ou ainda pela oportunidade de discutir sobre um grave problema de saúde pública mundial, a leitura do texto destaca a necessidade de programas, ações e mecanismos que visem aumentar a capacidade de aprendizagem, entendimento e atuação de familiares e cuidadores quanto aos cuidados de prevenção^(1)^. Apesar de todos os esforços, as atualmente denominadas lesões não intencionais ainda carecem de mais atenção devido ao elevado impacto sobre a saúde das crianças e às consequências sociais e econômicas tanto para a família quanto para toda a comunidade, visto que são uma destacada causa de sequelas, risco aumentado de incapacidades permanentes e morte^(2,3)^.

A ampliação do estudo das características epidemiológicas das lesões não intencionais e o conhecimento das repercussões sobre o contexto social têm contribuído muito para a implantação de estratégias de prevenção que incorporem novas ferramentas, como programas interativos e inteligência artificial, que facilitem a educação como meio de reconhecimento, ensinando medidas de primeiros socorros e, principalmente, ações preventivas que visem tanto atuar sobre o ambiente e seus desafios, modificando-os quando necessário, quanto orientar maneiras de adaptar o comportamento humano diante de cada situação^(4–6)^.

Além disso, aumentar o conhecimento e a conscientização entre pais e cuidadores sobre as características das lesões, facilitar o entendimento de seus mecanismos de atuação^(7,8)^, reconhecer o impacto sobre a saúde física, emocional e o desenvolvimento infantil são possibilidades reais que os games podem oferecer à comunidade, ensinando, capacitando e ajudando a desenvolver e aprimorar habilidades que possam ser direcionadas à maior segurança e aos cuidados com as crianças, sempre considerando os diferentes contextos sociais e culturais^(9)^.

# Translation: Seguridad del niño: una prioridad al alcance de todos

**DOI:** 10.1590/1980-220X-REEUSP-2025-0490es

**Authors:** Luiz Antonio Del Ciampo, Ieda Regina Lopes Del Ciampo

**Affiliations:** 1 Universidade de São Paulo, Faculdade de Medicina de Ribeirão Preto, Departamento de Puericultura e Pediatria, Ribeirão Preto, SP, Brasil.

Estimado Editor

El excelente y muy oportuno artículo publicado por Mendes *et al*. en esta Revista, cuyo estudio tuvo como objetivo crear y validar una aplicación móvil de *serious game* para apoyar la educación de los acompañantes sobre acciones de seguridad del paciente pediátrico, dirigida a los acompañantes de niños, invita a reflexionar sobre una de las cuestiones más importantes en salud: la seguridad del niño. Ya sea por el desarrollo de una aplicación móvil de *serious game* o por la oportunidad de debatir un grave problema de salud pública mundial, la lectura del texto pone de relieve la necesidad de programas, acciones y mecanismos orientados a fortalecer la capacidad de aprender, comprender y actuar de familiares y cuidadores en materia de prevención^(1)^. A pesar de todos los esfuerzos, las denominadas lesiones no intencionales aún requieren mayor atención debido a su elevado impacto en la salud infantil y a sus consecuencias sociales y económicas tanto para la familia como para la comunidad, ya que constituyen una causa importante de secuelas, mayor riesgo de discapacidades permanentes y muerte^(2,3)^.

El avance en el estudio de las características epidemiológicas de las lesiones no intencionales y el conocimiento de sus repercusiones en el contexto social han contribuido de manera significativa a la implementación de estrategias de prevención que incorporen nuevas herramientas, como programas interactivos e inteligencia artificial, capaces de favorecer la concienciación y el reconocimiento de riesgos, enseñar medidas de primeros auxilios y, principalmente, promover acciones preventivas orientadas tanto a intervenir sobre el entorno y sus desafíos, modificándolo cuando sea necesario, como a orientar sobre cómo adaptar el comportamiento humano ante cada situación^(4-6)^.

Los juegos pueden contribuir a aumentar el conocimiento y la concienciación entre padres y cuidadores sobre las características de las lesiones, facilitar la comprensión de sus mecanismos^(7,8)^ y favorecer el reconocimiento de su impacto en la salud física y emocional y en el desarrollo infantil. De este modo, pueden enseñar, capacitar y ayudar a desarrollar y perfeccionar habilidades orientadas a una mayor seguridad y a mejores cuidados de los niños, considerando siempre los diferentes contextos sociales y culturales^(9)^.
